# In Carpal Tunnel Syndrome, Sensory Nerve Conduction Velocities Are Worst in the Middle Finger Than in the Index Finger

**DOI:** 10.3389/fneur.2022.851108

**Published:** 2022-03-11

**Authors:** Kaoru Tada, Atsuro Murai, Yuta Nakamura, Yusuke Nakade, Hiroyuki Tsuchiya

**Affiliations:** ^1^Department of Orthopaedic Surgery, Graduate School of Medical Science, Kanazawa University, Kanazawa, Japan; ^2^Department of Clinical Laboratory, Kanazawa University Hospital, Kanazawa, Japan

**Keywords:** carpal tunnel syndrome, median nerve, nerve conduction study (NCS), middle finger, index finger

## Abstract

Although the index finger is generally used for sensory nerve conduction study in cases of carpal tunnel syndrome, there are reports that the middle finger should be used. The purpose of this study was to compare the results of sensory nerve conduction studies of the index finger and middle finger in patients with carpal tunnel syndrome. Among the 120 hands of 93 patients who were diagnosed with carpal tunnel syndrome and underwent carpal tunnel release surgery at our hospital, 54 hands of 48 patients who showed waveforms in sensory nerve conduction studies both index and middle fingers were included. 6 hands of 6 patients who showed no waveform in the index or middle finger, and 60 hands of 39 patients who showed no waveform in both index and middle finger were excluded. The subjects were 14 males and 34 females, and their ages were 66.2 years. The preoperative sensory nerve action potential (μV) and sensory nerve conduction velocity (m/s) of the index and middle fingers were tested using Wilcoxon's signed rank test. Spearman's rank correlation coefficient was also calculated for the results of the index and middle fingers. Sensory nerve action potentials were 2.0 in the index finger and 1.8 in the middle finger, with significantly lower in the middle finger. Sensory nerve conduction velocity was 30.1 in the index finger and 27.2 in the middle finger, with significantly lower in the middle finger. The correlation coefficients of sensory nerve action potentials and conduction velocities between the index finger and middle finger were 0.82 and 0.96, respectively, both of which showed a significant correlation. The results of the sensory nerve conduction studies of the middle finger were significantly worse than those of the index finger in cases of carpal tunnel syndrome. In addition, there was a strong correlation between the results of the index finger and the middle finger. The results of this study suggest that the nerve bundle to the middle finger may be more strongly affected than the nerve bundle to the index finger in cases of carpal tunnel syndrome.

## Introduction

Although the index finger is generally examined for sensory nerve conduction study in patients with carpal tunnel syndrome, there is some report that the middle finger should be examined (1). There have been reports of subjective evaluation and comparison of numbness and sensory disturbance in the index and middle fingers, but few reports of objective evaluation by electrophysiological study (2, 3). In this study, we compared the results of sensory nerve conduction studies of the index and middle fingers in patients with carpal tunnel syndrome.

## Materials and Methods

Among the 120 hands of 93 patients who underwent carpal tunnel release surgery by the same surgeon after diagnosis of idiopathic carpal tunnel syndrome based on clinical symptoms, physical findings, and results of electrophysiological tests, 54 hands of 48 patients who showed waveforms in sensory nerve conduction studies of both the index and middle fingers were included in this study. 6 hands of 6 patients who showed no waveform in the index or middle finger, and 39 hands of 60 patients who showed no waveform in both index and middle finger were excluded. The subjects were 14 males and 34 females, and their ages were 66.2 (58–71) [median (interquartile range)] years.

Sensory nerve conduction studies were performed by orthodromic stimulation with stimulating electrodes placed on the index and middle fingers and recording electrodes placed on the wrist joint with NeuroPack X1 (Nihon Kohden, Tokyo, Japan). Action potentials (μV) and conduction velocities (m/s) were measured by averaging 5–20 times (duration 0.2 ms, amplitude 10–20 mA). The distance between the stimulating and recording electrodes was measured each time. The room temperature was adjusted so that the surface temperature of the hand was above 32°C during the measurement.

The obtained action potentials and conduction velocities of the index and middle fingers were tested at a significance level of 5% using Wilcoxon's signed rank test. Additionally, Spearman's rank correlation coefficient was also calculated for the results of the index and middle fingers.

This research has been approved by the Institutional Review Board of the authors' affiliated institutions. All procedures followed were in accordance with the ethical standards of the responsible committee on human experimentation and with the Helsinki Declaration, and that informed consent was obtained from each subject.

## Results

Sensory nerve action potentials were 2.0 (1.1–4.7) [median (interquartile range)] in the index finger and 1.8 (0.9–3.6) in the middle finger, with significantly lower in the middle finger (*p* < 0.05). Sensory nerve conduction velocity was 30.1 (25.4–35.3) in the index finger and 27.2 (23.8–33.0) in the middle finger, with significantly lower in the middle finger (*p* < 0.001). The correlation coefficients of sensory nerve action potentials and conduction velocities between the index finger and middle finger were 0.82 and 0.96, respectively, both of which showed a significant correlation (*p* < 0.001). All of the 6 excluded hands of 6 patients showed waveform in the index and no waveform in the middle finger.

## Discussion

Carpal tunnel syndrome is reported to cause sensory disturbance in the thumb to ring finger, but the degree and frequency of sensory disturbance and the results of sensory tests and electrophysiological tests have been reported to vary by finger.

Okinaga et al. surveyed the fingers that first became aware of numbness in 102 hands of 52 patients with carpal tunnel syndrome and reported that the middle finger and ring finger were the most commonly reported to be the first to become aware of numbness (20%). They also compared the action potential and conduction velocity of the palmar digital nerve toward the second interdigital space and the palmar digital nerve toward the third interdigital space and reported that the results of the palmar digital nerve toward the third interdigital space was significantly worse (4). Hasegawa et al. (5) also investigated the presence or absence of numbness in 91 hands of 91 patients with carpal tunnel syndrome and reported that most patients were aware of numbness in the middle finger and ring finger.

Macdonell et al. compared the results of sensory nerve conduction studies using orthodromic stimulation from the thumb to the ring finger in 55 hands of 34 patients with carpal tunnel syndrome, and reported that in 9 hands, the action potential of the middle finger was <10 μV, but the action potential of the index finger was >10 μV, and the opposite pattern was not observed. They also reported that in six hands, the conduction velocity of the index finger was within the normal range despite the delayed conduction velocity of the middle finger, and the reverse pattern was not observed (2). Elfar et al. evaluated the subjective symptoms of the fingers and the results of 2-point discrimination (2-PD) and Semmes-Weinstein monofilament test (SWMT) in 40 hands of 35 patients with carpal tunnel syndrome. They found that many patients with carpal tunnel syndrome had subjective symptoms of the middle finger, and the 2-PD and SWMT results of the middle finger were significantly worse than those of other fingers (6).

These reports suggest that there are many cases of numbness in the middle finger in carpal tunnel syndrome, and that the middle finger tends to have worse results in sensory and electrophysiological tests than the index finger. In this study, the middle finger showed significantly worse results in sensory nerve conduction tests than the index finger. In addition, all of the 6 excluded hands of 6 patients showed waveform in the index and no waveform in the middle finger, suggesting that the middle finger may have more advanced nerve damage than the index finger.

On the other hand, Wee et al. compared the results of sensory nerve conduction studies using antidromic stimulation of the index and middle fingers in 178 nerves in 102 patients with carpal tunnel syndrome, and reported that there was no significant difference between the results of the index and middle fingers in terms of either action potential or conduction velocity (3). This result is contradictory to the above-mentioned report and the results of the present study, but it is thought that the difference in testing method may have affected the results, because testing with antidromic stimulation is considered to be less accurate than testing with orthodromic stimulation, as it is prone to contamination of compound muscle action potentials.

The topography of the median nerve is thought to be the cause of the differences in the frequency of numbness and test results between the fingers. Regarding the topography of the median nerve in the carpal tunnel, Sunderland's textbook states that the radial bundle of nerves to the thumb and the palmar digital nerve to the third interdigital space run in the shallow layer of the median nerve, the palmar digital nerve to the first interdigital space runs in its deeper layer, and the palmar digital nerve to the second interdigital space runs in its deeper layer (7).

Macdonell et al. (2) consider that the nerve bundle to the index finger runs deep in the median nerve and is less susceptible to compression from transverse carpal ligament. On the other hand, Okinaga et al. (4) consider that the palmar digital nerve to the third interdigital space runs superficial to the median nerve and is susceptible to compression from the transverse carpal ligament. Hasegawa et al. (5) also discussed the possibility that the neuropathy of the median nerve may develop from the palmar-radial lesion to the central lesion.

In addition, Akane et al. (8) evaluated SWMT and numbness of the fingers before and after carpal tunnel release surgery and reported that the middle finger was prone to residual numbness while SWMT results were likely to improve. This result suggests that the nerve bundle to the middle finger is more susceptible to compression injury and therefore more likely to have residual numbness, while recovery from decompression is more likely.

Currently, the index finger seems to be generally examined for sensory nerve conduction study in cases of carpal tunnel syndrome (9, 10). However, the results of this study showed that the results of the middle finger were significantly worse than those of the index finger, suggesting that if the purpose of sensory nerve conduction study is to detect nerve damage associated with carpal tunnel syndrome, the middle finger, which is thought to have more advanced nerve damage, might be useful for evaluation. However, in this study, there were 60 hands of 39 patients in which no waveform was detected from either the index or middle finger, accounting for about half of all cases. Even with the examination of the middle finger, it was considered difficult to assess the degree of nerve damage by sensory nerve conduction studies in cases with advanced carpal tunnel syndrome.

In conclusion, the results of sensory nerve conduction studies of the middle finger were worse than those of the index finger in cases of carpal tunnel syndrome. It was thought that the nerve bundle to the middle finger may be more strongly affected than the nerve bundle to the index finger in cases of carpal tunnel syndrome.

## Data Availability Statement

The raw data supporting the conclusions of this article will be made available by the authors, without undue reservation.

## Ethics Statement

The studies involving human participants were reviewed and approved by Research Ethics Committee, Graduate School of Medical Science, Kanazawa University. The patients/participants provided their written informed consent to participate in this study.

## Author Contributions

KT: conceptualization, writing—original draft, and project administration. AM and KT: methodology. YNakad and YNakam: investigation. HT: writing—review and editing and supervision. All authors contributed to the article and approved the submitted version.

## Conflict of Interest

The authors declare that the research was conducted in the absence of any commercial or financial relationships that could be construed as a potential conflict of interest.

## Publisher's Note

All claims expressed in this article are solely those of the authors and do not necessarily represent those of their affiliated organizations, or those of the publisher, the editors and the reviewers. Any product that may be evaluated in this article, or claim that may be made by its manufacturer, is not guaranteed or endorsed by the publisher.
